# Critical transitions in a game theoretic model of tumour metabolism

**DOI:** 10.1098/rsfs.2014.0014

**Published:** 2014-08-06

**Authors:** Ardeshir Kianercy, Robert Veltri, Kenneth J. Pienta

**Affiliations:** Brady Urological Institute, Johns Hopkins Hospital, Baltimore, MD 21287, USA

**Keywords:** tumour metabolism, game theory, Warburg effect, epithelial–stromal metabolic decoupling, metabolic symbiosis in cancer, lactate shuttle

## Abstract

Tumour proliferation is promoted by an intratumoral metabolic symbiosis in which lactate from stromal cells fuels energy generation in the oxygenated domain of the tumour. Furthermore, empirical data show that tumour cells adopt an intermediate metabolic state between lactate respiration and glycolysis. This study models the metabolic symbiosis in the tumour through the formalism of evolutionary game theory. Our game model of metabolic symbiosis in cancer considers two types of tumour cells, hypoxic and oxygenated, while glucose and lactate are considered as the two main sources of energy within the tumour. The model confirms the presence of multiple intermediate stable states and hybrid energy strategies in the tumour. It predicts that nonlinear interaction between two subpopulations leads to tumour metabolic critical transitions and that tumours can obtain different intermediate states between glycolysis and respiration which can be regulated by the genomic mutation rate. The model can apply in the epithelial–stromal metabolic decoupling therapy.

## 1. Introduction

Tumours are ecosystems that consist of different phenotypic cell populations. Metabolic dynamics within a tumour build on these population interactions [1–5]. Tumour cells reprogramme their metabolism and cooperate with each other to meet the challenge of uncontrolled proliferation. Empirical observations show that cancer cells and stromal fibroblasts dynamically co-evolve and become metabolically coupled [6]. The co-evolving dynamics of this metabolic symbiosis is the subject of this study.

Tumour cells alter their metabolic patterns compared with those of normal cells and use both glycolysis and respiration [7]. This dynamic alteration in tumour metabolism is regulated by intracellular mechanisms such as oncogenes, tumour suppressor genes [7,8] and the genomic instability rate [9].

Furthermore, it has been suggested that separating intracellular pathways and intercellular interaction fails to capture a realistic pattern of tumour metabolism. Indeed, in most tumours both the cancer-producing pathways (e.g. p53, AKT, NFκB, C-MYC and mTOR) and intercellular communication network evolve in tandem. However, intercellular signalling such as lactate secretion in some tumour microenvironments still needs more careful study and thus has attracted significant interest in recent years [6,10–14]. Our game-driven model looks at intercellular *lactate-shuttle* interaction between epithelial cancer cells and stromal fibroblast cells and proposes an evolving tumour ecosystem based on the notion of interacting adaptive cells.

Adenosine triphosphate (ATP) production pathways have been a subject of game theoretical approaches [15–18], and cooperation in tumour metabolism can be put in the framework of game theory [5,19,20]. Here, we extend these previous studies by providing a model which is based on collective evolution of adapting tumour cells while the selection pressure is regulated by both the amounts of available energy (ATP) and the degree of the genomic instability rate of the tumour.

We use a framework defined by replicator dynamics equations [21,22] which contain an exploration term to capture the genomic instability of cancer. We study the behaviour of the dynamics and its fixed point stability at different genomic mutation rates over energy generation pathways. The model captures the relationship between the mutation rate of the tumour and glycolysis–respiration transitions. Understanding the metabolic transitions in the tumour and the role of lactate consumption in the oxygenated cancer cell metabolism may suggest new non-toxic therapeutic strategies based on uncoupling the stromal–epithelial interactions in adenocarcinomas.

## 2. Tumour metabolic coupling

In 1926, Warburg *et al*. [23] addressed a metabolic abnormality in cancer cells in which cancer cells under normal oxygen concentrations switch from aerobic energy generation through oxidative phosphorylation to anaerobic energy generation through glycolysis, using glucose for energy production without oxygen. This metabolic shift forms the basis of the *Warburg effect* [24,25].

**Figure 1.**
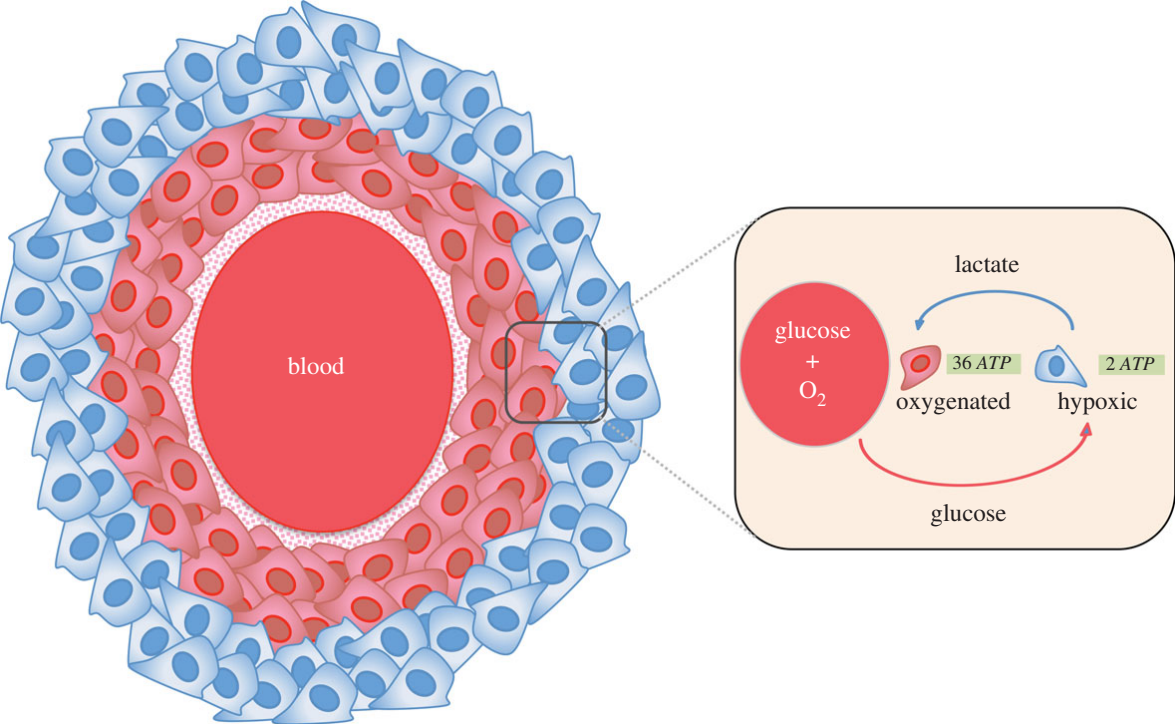
Tumour metabolic symbiosis between oxygenated and hypoxic cells. Glucose and lactate are energy sources used in hypoxic and oxygenated cells, respectively, which is the Nash equilibrium of the metabolic symbiosis game. In the Nash equilibrium hypoxic cells generate 2 mol of ATP and 2 mol of lactate per mole of glucose, whereas aerobic cells generate 36 mol of ATP per 2 mol of lactate. (Online version in colour.)

However, recent studies have demonstrated that the Warburg effect only characterizes a portion of the tumour metabolism happening in the hypoxic domain of the tumour. Some hypoxic cells use glycolysis and produce lactate as a metabolic product whereas oxygenated cells use the lactate to generate ATP (figure 1). Thus, a tumour can use the lactate from glycolysis as a source of energy through a lactate shuttle between hypoxic and oxygenated cell populations [10–13]. This phenomenon forms the *reverse Warburg effect* [6].

This metabolic symbiosis, which promotes tumour progression, is often observed between cancer cells and stroma [26–32]. For example, in prostate cancer the presence of metabolic symbiosis between epithelial cancer cells and their associated fibroblasts promotes a high Gleason score and tumour progression to metastasis [31,33]. Next, we define a normal game for the metabolic coupling between these two subpopulations in a tumour.

## 3. A metabolic symbiosis game

Evolutionary game theory provides a systematic explanation for tumour structure as an outcome of the cellular decision process [2,34]. Strategic decisions on the utilization of energy resources is necessary for handling limitations based on available nutrients and oxygen in the tumour. A cell can be considered as an agent which responds to changing environmental conditions by changing its strategy based on the energy uptake sources. This decision process can be placed in the framework of evolutionary game theory.

To apply this theory to tumour metabolism coupling requires definition of a game that includes players and actions in tumour metabolism. The metabolic symbiosis between the two subpopulations can be put in the framework of a two-player, two-action normal game. The tumour microenvironment roughly consists of two main domains, one domain is adjacent to the vascular system (the oxygenated cell population) and the other domain has less available oxygen (the hypoxic cell population). Hypoxic and oxygenated cell populations are the two players in a normal game.

It is known that cells can use different sources of energy such as fatty acids, glutamine [35], monocarboxylic acids (like lactate) and glucose. But for simplicity, we considered only the two well-studied cancer cell energy sources, glucose and lactate. Thus, each cell (player) can select different energy metabolic pathways (actions) which are suited to the use of glucose or lactate.

The reward matrix of the game is based on the ATP per mole production, which reflects the cellular metabolic rate. It is known that the oxygenated cells—not the hypoxic cells—can use lactate for energy generation, the value of which is defined as *L*. The energy production by oxygenated or hypoxic cells using glucose is indicated by *G*_o_ or as *G*_h_, respectively. If two oxygenated or hypoxic cells meet while both are using glucose, then the glucose energy source divides between each of them as *G*_o_/2 or *G*_h_/2, respectively. It is also assumed that the only source of the lactate in the tumour is provided by the hypoxic cells' glycolysis process.

**Figure 2.**
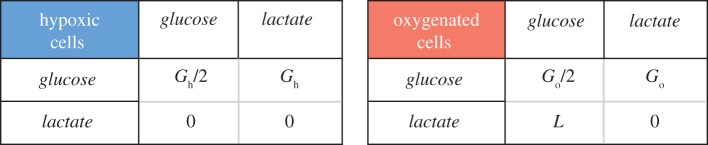
The 2 × 2 reward (ATP generation) matrices for the tumour metabolic symbiosis as a two-player, two-action game. The left matrix represents hypoxic, and the right one represents oxygenated cell energy generation values based on their collective actions. Empirical data show that *L* > *G*_o_/2. (Online version in colour.)

The general reward matrix for the two-player, two-action tumour metabolism game is shown in figure 2.

The Nash equilibrium is a central concept in game theory. A strategy profile forms a Nash equilibrium if no player can increase its expected reward by *unilaterally* deviating from the equilibrium. This means that neither of the two subpopulations can gain more energy by cheating and changing their strategy unilaterally.

Empirical data demonstrate that *L* > *G*_o_/2 [11,36], and therefore the symbiosis game has only one pure Nash equilibrium, with a corresponding energy production of *L* for oxygenated cells and *G*_h_ for hypoxic cells (figure 2). In other words, the Nash equilibrium of the game corresponds to the observed tumour metabolic symbiosis.

The prediction of the game model is that tumour cells converge to the Nash equilibrium where hypoxic cells only use glycolysis for energy generation. In reality, hypoxic cells do not fully transform their energy metabolism to the glycolytic state, but change to a range of glycolysis metabolism levels [36,37]. To address this deviation from complete symbiosis, we propose a dynamic schema that can obtain different intermediate stable fixed points to represent the hybrid metabolism of glycolysis and lactate-fuelled respiration.

## 4. Evolutionary dynamics

The crosstalk between cell proliferation signal transduction and cellular energy metabolism is critical for cell survival in a changing environment. Cell energy metabolism alterations usually come with changes in cellular proliferation and vice versa [36,38]. To capture this dependency in a simple mathematical formula, it is assumed that the replication capacity of a cell is a Boltzmann function of the cell energy generation. It is assumed that the Boltzmann function can capture the nonlinear relation between energy production and cellular proliferation [39,40]. We assumed that the probability *x_i_* of selecting metabolic pathway *i* with metabolic rate *r_i_* is given by4.1
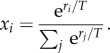
*T* is a positive parameter^1^ which controls the trade-off between exploration and exploitation in the space of cellular energy pathway options: for *T* → 0 tumour cells choose the strategy corresponding to the maximum energetic value (pure exploitation), whereas for *T* → ∞ the cell's strategy is completely random (pure exploration). The tumour cells demonstrate a wide range of exploration in phenotypic states. The *T*-value changes during different stages of cancer development or at different stages of therapy. Genetic and epigenetic mutations are among the possible cellular biological factors which can determine the *T*-value.

*Applying the genomic mutation rate in the evolutionary modelling.* Tumorigenesis can promote by abnormality in mutation rates within the genome. For example, in hereditary cancers genomic instability is already present in precancerous tissues [9], which leads to tumour development by increasing the mutation rate, thus increasing the value of the cellular exploration rate *T*.

To address this cancer hallmark, our model assumes that the *T*-value reflects the rate of function-altering mutations and genomic instability during tumour progression. There is usually an increase in the genetic/epigenetic mutation rate during the progression of carcinomas [9]. Many of the tumorigenesis mutations target the DNA-maintenance machinery. As a result of failure in the DNA maintenance the tumour microsatellite instability (MSI) increases. Thus, one possible way to evaluate *T* is to assess tumour MSI and length distribution of the microsatellite.

### 4.1. Dynamic modelling

Here, we are specifically focused on the temporal evolution of tumour states. Let *i* be the phenotype with the energy pathway that uses energy source *i* where *i* = 1,2, … ,*n*, and *r_i_* is the observed metabolic rate that has a direct proportional relation with ATP production through pathway *i* and frequency of population or strategy *i* is *x_i_*, then it has been proved [22] that the following dynamics fixed points are in the form of the Boltzmann distribution (equation (4.1)):4.2
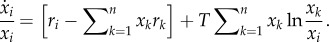


The dynamics explains how strategic optimization is reached in an ecosystem through cell proliferation success. The first term in equation (4.2) demonstrates that the probability of selecting energy metabolism *i* increases with a rate proportional to the overall efficiency of that strategy, whereas the second term describes the cells' tendency to *explore* across possible energy pathways.

Now assume there are two types of populations *X* and *Y* that are adapting concurrently. Let *A* and *B* be the two reward matrices: *a_ij_* and *b_ij_* are the rewards of the *X* and *Y* population when one selects *i* and the other population selects *j*. This reward can be in the form of biological pay-off, such as the cellular metabolic rate. Furthermore, let **x** = (*x*_1_, … ,*x_n_*), 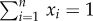 and **y** = (*y*_1_, … ,*y_n_*), 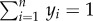 be the strategy of the first and the second population, respectively.

The learning dynamics in a two-player scenario case is obtained from equation (4.2) by replacing *r_i_* with the expected pay-off using the reward matrix, which yields4.3
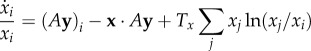
and4.4
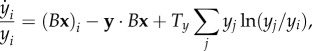
where (*A***y**)*_i_* and (*B***x**)*_i_* are the *i* element of the vectors *A***y** and *B***x** that determine the expected rewards of the population *X* and *Y*, respectively. *T*_*x*_ and *T*_*y*_ represent the exploration rates in the metabolic pathways of population *X* and *Y*, respectively.

It is important to note that the stable rest points for replicator system equations (4.3) and (4.4) at *T_x_* = *T_y_* = 0 are also the Nash equilibrium of a game [41]. One can speculate that higher genetic and epigenetic mutation rates lead to a higher *T*-value. We study the outcomes of the dynamical system equations (4.3) and (4.4) when *T_x_* and *T_y_* are not zero.

To examine a typical metabolic environment, empirical data were used from studies that report typical values of ATP production in the tumour oxygenated and hypoxic cells, using either lactate or glucose for energy metabolism [10,36,37].

Substituting these empirical values in the reward matrix in figure 2 provides a typical example of the tumour metabolic symbiosis game reward matrices. A typical reward matrix for the hypoxic cells *A* is4.5
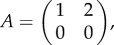
and for the oxygenated cells *B* is4.6
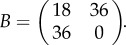
In both matrices, the first and the second action is choosing glucose and lactate, respectively, as the energy source.

## 5. Critical transitions in tumour metabolism

Critical transitions describe sudden changes in the outcome of dynamical systems when an underlying process parameter slightly changes. Many natural ecosystems may suddenly switch to different stable states [42] as a result of critical transitions. Here, we consider the tumour microenvironment as an evolving ecosystem and investigate the role of genomic instability and tumour metabolic symbiosis in leading the tumour to intermediate states between lactate respiration and glycolysis. This can be a sign of metabolic critical transitions in the tumour's evolving ecosystem. Nonlinearities in the interactions between hypoxic and oxygenated cells, together with the intrinsic sensitivity of cell proliferation to the energy variation, provide rich dynamics, which can lead to alternative stable states in tumour energy metabolism.

**Figure 3.**
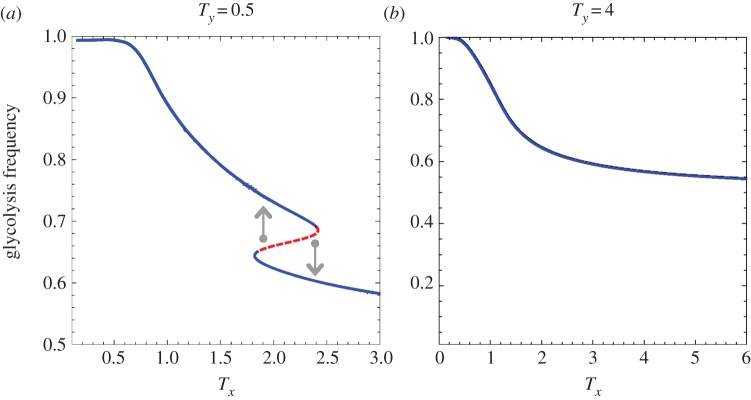
Transitions in the stability of hypoxic cells' metabolic states. From left to right, the value of * T_y_* increases. (*a*) Bistability in the hypoxic cells population and (*b*) high glycolysis as a globally stable state in the hypoxic cells. The stable (solid blue line) and unstable (dashed red line) outcomes of the tumour metabolic symbiosis game change as *T* values cross over critical values. The arrows indicate the direction of change from the dashed unstable state to the two alternative stable states on the upper and lower branches. The dashed red line marks the border of the two stable state basins of attraction. (Online version in colour.)

**Figure 4.**
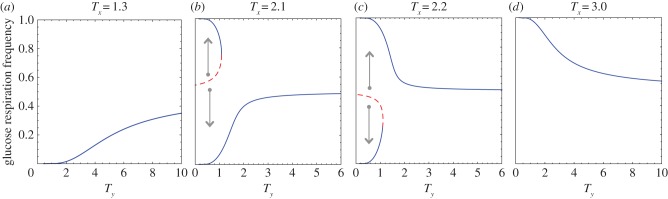
Transition in the stability of oxygenated cells' metabolic states. From left to right, the value of *T_x_* increases. (*a*) High lactate uptake by oxygenated cancer cells as a global equilibrium. (*b*,*c*) Bistabilty in the oxygenated population. (*d*) High glucose respiration in oxygenated cells becomes the global stable equilibrium. The stable (solid blue line) and unstable (dashed red line) outcomes of tumour metabolic symbiosis dynamics change as *T*-values cross over critical values. The arrows indicate the direction of change from the dashed unstable state to the two alternative stable states on the upper and lower branches. The dashed red line marks the border between the two stable state basins of attraction. (Online version in colour.)

Equations (4.3) and (4.4) show critical transitions with regard to *T_x_* and *T_y_* values. The bifurcation graphs in figures 3 and 4 represent the tumour glycolytic and lactate-fuel respiration level at equilibrium with regard to the cellular exploration rates.

There is a critical range of *T_x_* and *T_y_* where there are two stable equilibrium states, separated by an unstable equilibrium that marks the border of the *basin of attractions* between the two stable states. Outside of the critical range the tumour ecosystem is in a global stable equilibrium state. Bifurcation graphs in figures 3 and 4 illustrate this critical transition of the dynamics for both hypoxic and oxygenated populations.

*Critical transitions in hypoxic tumour cells.* Figure 3 illustrates that hypoxic cells mainly use glycolysis, regardless of *T*-values. Above a critical value of *T_y_*, the hypoxic cells always stay in the upper branch of the bifurcation (figure 3*b*), which corresponds to high glycolysis.

*Critical transitions in oxygenated tumour cells.* Figure 4 demonstrates that the oxygenated cell population in the tumour ecosystem can shift between two levels of lactate-fuel respiration. At very low *T_x_* (figure 4*a*), oxygenated cancer cells are in the lower branch with high lactate respiration. In a certain range of *T*_*x*_ the dynamics have bistable equilibrium (figure 4*b,c*).

Finally, in figure 4*d*, oxygenated cancer cells mainly use glucose respiration. In this case, the hypoxic cells might lose their survival advantage in the tumour.

## 5.1. Different levels in tumour metabolic symbiosis

**Figure 5.**
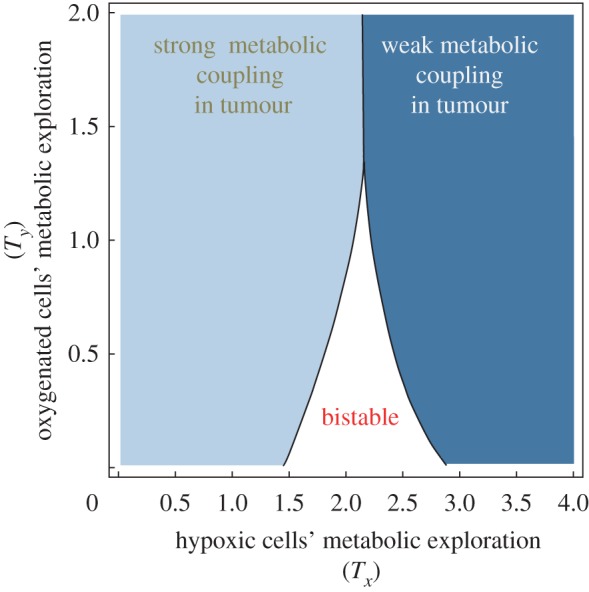
Characterization of different levels of the tumour metabolic symbiosis in the parameter space with (*T_x,_T_y_*). The solid dark region corresponds to cells that can have one stable state. The border between different zones corresponds to the critical values of *T*. (Online version in colour.)

The model shows that metabolic transitions lead to different classes of tumour with possibly different therapeutic plans. The critical values of *T_x_* and *T_y_* are not independent. A domain of parameters (*T_x_* and *T_y_*) demonstrate bistability in the dynamics equilibrium. This leads to different zones in the parameter space of *T_x_* and *T_y_*. Figure 5 demonstrates different categories of tumour cells based on their exploration rates in the energy production strategy space, i.e. *T_x_* and *T_y_*.

We consider a tumour metabolic state as a *strong metabolic coupling*, if at least half of the tumour cells participate in metabolic symbiosis, and we consider it a *weak metabolic coupling* if less than half of the tumour cells participate in metabolic symbiosis. In the bistable domain of figure 5, the state of the tumour metabolic coupling can shift to different levels based on the tumour ecosystem population history (initial tumour population structure).

There is a domain of (*T_x_*, *T_y_*) in figure 5 which represents strong metabolic symbiosis. This indicates a strong coupling between tumour cells where cancer cells gain the advantage of growth and survival in the limiting nutrient resources.

### 5.2. Epithelial–stromal metabolic decoupling

Different types of cancer cells, including breast, ovarian and prostate, can secrete hydrogen peroxide, generating free radicals that then trigger oxidative stress in their neighbouring stromal fibroblasts cells [43]. Therefore, epithelial cancer cells activate stromal fibroblasts to secrete high levels of lactate and pyruvate that are used by tumour cells for ATP production via respiration. A metabolic stroma-specific therapeutic is suggested to target the metabolic coupling between these two type of subpopulations in the tumour microenvironment [44,45]. Here, we examine potential hints from our model for a stromal-specific therapeutic [44] in adenocarcinomas.

#### 5.2.1. The effect of genomic instability rate

The model shows that stromal and epithelial cancer cells explorations rate in the metabolic pathways, *T_x_* and *T_y_*, respectively, are important for a therapeutic intervention plan.

**Figure 6.**
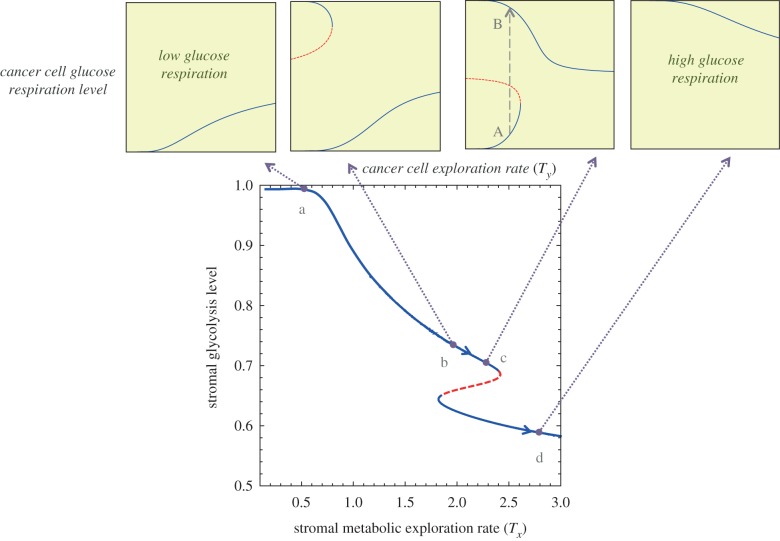
Different epithelial–stromal decoupling outcomes. Line AB corresponds to a therapeutic path which targets the lactate shuttle and shifts the cancer cells to a high glucose respiration state B, while the stromal cells also mainly consume glucose. This leads to a weak tumour metabolic symbiosis. (Online version in colour.)

Figure 6*a* shows that in that stage of the tumour there is only one theoretical state of the tumour where stromal cells provide the main source of energy for epithelium cancer cells. In figure 6*d*, the tumour has again only one theoretical state where stromal cells gradually show less secretion of lactate. However, the tumour metabolic dynamical behaviour is more complex in the intermediate cases such as those in figure 6*b,c*.

In the case in figure 6*b*, the cancer epithelial cells can obtain two stable states. The cancer cells with high glucose uptake can go through a sudden critical transition to a high lactate uptake state. However, a small perturbation in the stromal metabolism is enough to shift those cases such as figure 6*b* to a completely opposite tumour metabolic state such as figure 6*c*. In the case of figure 6*c*, targeting the lactate shuttle can push the cancer cell population structure to a higher glucose uptake state, thus resulting in a weak tumour metabolic symbiosis.

#### 5.2.2. Targeting the lactate shuttle

Increased expression of lactate monocarboxylate transporters (MCTs) and activation of the lactate respiration pathway are common aberrations in adenocarcinomas. Epithelial cancer cells induce the surrounding stroma to express MCT4 for lactate efflux. The level of metabolic cooperation can be tested by measuring the lactate transportation between cells that is mediated by MCTs, especially MCT1 and MCT4, which are highly expressed in cancer cells. For example, in both oestrogen receptor-positive and oestrogen receptor-negative breast cancer tumours, the high MCT4 expression is associated with more aggressive tumours. Furthermore, cancer epithelial cells upregulate the expression of MCT1 as a transporter for lactate uptake [28].

The lactate dehydrogenase-A (LDH-A) enzyme catalyses the conversion of pyruvate and lactate, and its upregulation is associated with aggressive tumour outcomes. Emperical results show that tumour cells are susceptible to LDH-A inhibition [46]. Thus, LDH-A can be a viable therapeutic target for disturbing the lactate shuttle. Moreover, it is suggested that it may be unwise to use lactate-containing intravenous solutions such as lactated Ringer's or Hartmann's solution in cancer patients [47].

Any method of lactate downregulation such as MCT1,4 or LDH-A inhibition therapies can be designed for the tumour state similar to the case in figure 6*c*, where the cancer cell population can alter from a high lactate uptake state to a high glucose respiration state, thus the therapeutic outcome would be a sudden decrease in tumour cancer cell metabolic coupling which can lead to decreased tumorigenesis. Using the bifurcation graphs in figure 6, the therapy can be designed for an effective intervention plan and proper dosage of adjuvant radio/chemotherapy.

## 6. Conclusion

This study applied the mathematical formalism of evolutionary game theory to demonstrate that cancer metabolism might show critical transitions over certain ranges of biological conditions. We used the Warburg effect and reverse Warburg effect to define a tumour metabolic game between oxygenated and hypoxic tumour cells. Dynamic modelling results show the coexistence of two stable states (bistability) in tumour ecology. Our model shows that a simple intercellular signal such as lactate secretion in some tumour microenvironments can induce a critical transition between high and low levels of tumour glucose consumption. Our co-evolving dynamics also can classify different tumour cells, which provides useful hints during stromal–epithelial cancer cell metabolic decoupling therapy.

We acknowledge that a Boltzmann-like mechanism is a simplified version of the cell behaviour, but it can be incorporated into experimental designs for future studies on the interaction between cellular energy and proliferation pathways. One of our model limitations is the assumption of the ecosystem with two different phenotypic cells—hypoxic and oxygenated—as *a priori*. In addition, we assumed that the glucose level, lactose level and blood vasculature remain constant and the hypoxic–oxygenated population changes only by intramural interactions. Therefore, one can extend this work by investigating the metabolic changes as a result of glucose/lactate level variations, considering lipid metabolism and also blood vasculature development—angiogenesis—in the tumour.

In addition, lactic acid can also be detrimental to the cancer cells. In this study, we consider the lactate acid contribution to the oxygenated cells as an energy fuel. One can also consider the detrimental role of the lactate, as the lactate acid concentration passes a threshold value and becomes toxic for the neighbouring cells. On the other hand, a threshold proportion of lactate secretion might be needed before the emergence of functional symbiosis.

This study shows that tumour cells in certain ranges of metabolic exploration rate can obtain alternative stable states. Our model prediction is consistent with empirical data on the mixed energy metabolism of cancer cells. Breaking the metabolic coupling in tumours may have therapeutic benefits. However, one of the major challenges is to find a *therapeutic window* for sensitive tumour cells [29,48]. To this end, this study distinguishes certain types of cancer cells that are more vulnerable to a metabolic uncoupling anti-cancer therapy.

## Endnote

1 It resonates with Robert H. Austin's [49] mechanistic relation between proliferation and fitness of a population in an evolving ecology.
